# Evaluation of optical genome mapping for detecting chromosomal translocation in clinical cytogenetics

**DOI:** 10.1002/mgg3.1936

**Published:** 2022-04-06

**Authors:** Peng Dai, Xiaofan Zhu, Yanzheng Pei, Peng Chen, Jingjing Li, Zhi Gao, Yu Liang, Xiangdong Kong

**Affiliations:** ^1^ The Genetics and Prenatal Diagnosis Center, The Department of Obstetrics and Gynecology the First Affiliated Hospital of Zhengzhou University Zhengzhou China; ^2^ GrandOmics Diagnostic Wuhan China; ^3^ Department of Neurology The First Hospital of Yulin Yulin China

**Keywords:** chromosomal translocation, CNV‐seq, cytogenetics, FISH, karyotyping, optical genome mapping

## Abstract

**Background:**

Balanced reciprocal translocation is one of the most common chromosomal abnormalities in humans that may lead to infertility, recurrent pregnancy loss, or having children with physical or mental abnormalities. Karyotyping and FISH are traditional detection approaches with a low resolution. Bionano optical genome mapping (OGM) developed in recent years can be used to analyze chromosomal abnormalities at a higher resolution, providing the possibility of more in‐depth analyses of balanced chromosome translocations.

**Methods:**

To evaluate the feasibility of OGM to detect chromosome balanced translocations, 10 genetic outpatients were collected and detected simultaneously by karyotype analysis, FISH, CNV‐seq, and Bionano OGM in this study.

**Results:**

The results showed that the karyotypes of the patients were detected by karyotype analysis, FISH, and Bionano OGM, but one patient with karyotype *t*(Y,19) was not correctly detected by OGM. There were not find any chromosome abnormality by CNV‐seq. More importantly, OGM allowed the location of the mutation to the gene level, which is important for aiding diagnoses, compared to karyotype analysis, and FISH.

**Conclusions:**

This study shows that OGM can be a high adjunctive diagnostic method for detecting balanced chromosome translocations, but the accuracy and precision of OGM detecting mutations need to be gradually improved in telomere and centromere regions.

## Graphical abstract

Our study shows that optical mapping technique can be a high adjunctive diagnostic method after karyotyping and FISH for detecting balanced chromosome translocations.

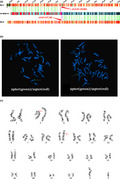

## 1 BACKGROUND

Balanced reciprocal translocations are common chromosomal structural rearrangements, formed by random de novo breakage and rejoining of two or more chromosomes (Wang et al., 2020). It occurs in approximately 0.2% of the human population and 2.2% in patients who experience a history of recurrent miscarriages (Jacobs et al., 1992). Carriers with balanced reciprocal translocations could be phenotypically normal while carrying fertility problems, such as infertility, early abortion, fetal death, and malformations (Chantot‐Bastaraud et al., 2008; Hofherr et al., 2011), due to the prevalence of producing unbalanced gametes (Chow et al., 2020). The proportion of resulting unbalanced embryos can be highly variable depending on the sex of the carriers, the type of chromosomes involved, and the positions of the breakpoints (Chow et al., 2020). Therefore, detection of phenotypically normal carriers of balanced reciprocal translocations has obvious clinical implications for guiding fertility and preventing the birth of children with genetic defects.

Traditional chromosome analysis techniques, such as karyotyping and fluorescence in situ hybridization (FISH), have been the basic prenatal and postnatal diagnostic methods and are capable of detecting most types of balanced and unbalanced chromosomal abnormalities, as well as numerical chromosomal aberrations in clinical genetics (Bates, 2011; Dremsek et al., 2021; MacLeod & Drexler, 2013; Speicher & Carter, 2005). Both of these techniques are not only time consuming and require a high level of manual proficiency, but also make it difficult to determine subtle changes at a relative low‐resolution level (Bates, 2011). Karyotyping can be used to locate abnormal chromosomal regions within a range of 5–10 Mb, while it is not conducive for precise analyses of the clinical consequences because a large number of genes may be inside the variant region. FISH is capable of detecting variants in the level of individual genes, but it is dependent on designed probes and is not applicable to highly accurate analyses at the genome‐wide level. Recent developments in microarray and next generation sequencing (NGS) technologies can be used to enable copy number variation (CNV) analyses at the whole genome level, while they lack the ability to detect chromosomal translocations or inversions (Hu et al., 2019; Weckselblatt & Rudd, 2015). Hence, it is essential to develop a more applicable method to complement the existing methods described above.

Bionano optical genome mapping (OGM) is a recently developed technique can directly image labeled DNA molecules. Endonucleases are used in Bionano OGM instruments to recognize DNA sequences and cut single strands, so as to simultaneously label fluorescence and subsequently straightening, linearizing, and unfolding each single DNA molecule by very fine capillary electrophoresis for high‐resolution fluorescence imaging of ultra‐long single molecules (Jeffet et al., 2021; Schwartz et al., 1993). The structure of chromosomes can be analyzed according to the order of fluorescence signals on DNA molecules. OGM was initially applied to de novo assembly of genomes, and the combination with NGS technique has allowed for a more complete genome assembly (Mostovoy et al., 2016; Sun et al., 2020). OGM has shown great advantages in the detection of genomic structural variants (Chan et al., 2018). Hence, in this study, OGM was performed to analyze 10 cases meanwhile compared with karyotype analysis, FISH as well as CNV‐seq, so as to explore its feasibility for clinical assistance in the diagnosis of balanced chromosome translocations.

## 2 MATERIALS AND METHODS

### 2.1 Samples and clinical data

A total of 10 patients with clinically affected fertility, including eight males and two females, were collected in this study at the First Affiliated Hospital of Zhengzhou University. These individuals had been previously analyzed through standard cytogenetic karyotyping, all whom carried unique reciprocal translocation. The details of patient demographics are shown in Table 1.

**TABLE 1:** The details of patient demographics

Sample	Gender	Years	Clinical symptoms	Karyotype
M18A0732	Male	29	Oligospermia	*t*(10;11)(p12.2;q12)
M18A1031	Male	27	Azoospermatism	*t*(2;22)(q22;q13)
M18A0915	Male	29	Azoospermatism	*t*(Y,19)(q11.21,q12)
M18A1012	Male	35	Embryo damage 1 time	*t*(8;15)(p11.2;q26.1)
M18A1329	Male	23	Spontaneous abortion 1 time	*t*(2;16)(q13,q12.1)
M18A1203	Male	23	Spontaneous abortion 1 time and embryo damage 1 time	*t*(2;9)(p22;p23)
M18A1077	Male	28	Spontaneous abortion 2 times	*t*(11;22)(q24;q12)
M18A1345	Female	23	History of abnormal birth on chromosome 9 and 21	*t*(9;21)(q21.13;q11.2)
M18A1052	Male	33	Oligospermia	*t*(3;14)(q21;q24)
M18A1399	Female	23	Primary infertility	*t*(2;13)(p11.2;q14)

### 2.2 Isolate DNA, DNA labeling, and chip loading

gDNA with an ultra‐high molecular weight (UHMW) was isolated and DNA labeling was done following the manufacturer's guidelines (Bionano Prep SP Frozen Human Blood DNA Isolation Protocol, Bionano Genomics #30246). Briefly, UHMW gDNA was isolated from frozen human blood using Bionano PrepTM Blood and Cell Culture DNA Isolation Kit (Bionano Genomics), and DNA was quantified by Qubit dsDNA assay BR kit with a Qubit 3.0 Fluorometer (ThermoFisher Scientific) for DNA quantification, and the DNA concentration should fall between 45 and 200 ng/μl. A total of 1 μg of UHMW DNA was labeled using DNA Labeling Kit‐NLRS (Bionano Genome). UHMW gDNA was fluorophore‐labeled at the cleavage sites of special restriction enzymes. Final quantification was carried out before loading the samples onto the Bionano Saphyr chip, with a recommended DNA concentration of 3–10 ng/μl. The labeled DNA was stained for backbone visualization, which was then loaded on Bionano chips. When the Bionano chip was run on the Saphyr instrument, images of the fluorophore‐labeled nicks and stained‐DNA backbones were captured while BNX files containing the original information about the DNA molecules as well as labels as the input of subsequent analyses.

### 2.3 Data collection

The labeled DNA was loaded on Bionano Saphyr Chip (Bionano Genome) and data were collected on the Saphyr instrument with hg38 genome for real‐time quality control assessment, with the goal of covering 100 × raw human genome.

### 2.4 De novo assembly and structural variants calling

De novo assembly and structural variants (SVs) calling were performed via a de novo assembly pipeline through Bionano Access software (v1.2.1). First, molecules were filtered by length and label density, following the default setting of an algorithm where molecules were longer than 150 kb, spanning at least nine labels per 100 kb and the signal‐to‐noise ratio should be higher than 2.75, while the maximum backbone intensity should be 0.6. Then, all filtered molecules were taken as input of the de novo assembly. Molecules were assembled to consensus genomes mapping with the following parameters: iterations = 5, de novo assembly noise, namely the specially false positive density (/100 kb) = 1.0, false negative rate (%/100) = 0.1, site sd = 0.12, scaling sd (kb/^1/2) = 0.0, relative ds = 0.016, resolution sd = 0.25, the cutoff threshold for the initial assemble parameter *p* value was 1e^−11^ and the extension and refinement *p* value was 1e^−12^, excepted for M18A1077 and M18A1399, which were of an optimized *p* value.

SVs were identified based on the alignments of the assembled genome with the human reference hg38 (GCA_000001405.15) using a multiple local alignment algorithm and inconsistent pairs of alignments representing potential SV events. Types such as insertions, deletions, inversion breakpoints, translocation breakpoints, and copy number variants (CNVs) were detected through this pipeline. It should be noted that translocation breakpoints within chromosomes were invoked when the inversion size was greater than 5 Mb.

### 2.5 Fluorescence in situ hybridization (FISH)

FISH was performed at Be Creative Lab (Beijing) Co. ltc. Fluorogenic‐labeled probes designed according to the events of karyotyping were prepared in lab, hybridized with standard cytogenetic cells, and chromosomes were counterstained with DNA‐specific fluorochromes such as DAPI. Finally, fluorescent signals were analyzed via a super‐resolution fluorescence microscope.

### 2.6 Copy number variations (CNVs)

CNVs were detected by next generation sequence as described (Liang et al., 2014; Trost et al., 2018; Wang et al., 2018). Typically, 200 ng DNA was fragmented, a 300 bp‐sequencing libraries were constructed and then sequenced on the illumina X10 platform (Illumina). The results were analyzed using previously described algorithms.

### 2.7 Data comparison

To assess the consistency of the structures detected through different methods, we performed multiple comparisons of the SVs detected by OGM, karyotyping, and FISH on the 10 patients. The identical SVs among these methods were delineated by chromosomes and bands on the p or q arm.

## 3 RESULTS

### 3.1 Optical genome mapping data

The 10 samples resulted in 3.2 Tb of data in total by OGM. The average data were 317 Gb (range 233–551 Gb). After filtering, the average N50 of those molecules was 272 kb (range: 172–388 kb), and the average label density was 12.7 per 100 kb (range: 10.6–16.1). The average mapping ratio mapped to reference genomes from molecules was 73% (range: 64%–80%), and the average effective coverage was 59 × (range: 41–83 ×), the above details are shown in Table 2.

**TABLE 2:** The details of OGM data

Sample	Total length (Gb)	Average length (kb)	Molecular N50 (kb)	Label density (/100 kb)	Effective coverage of reference (X)	Map rate (%)
M18A0732	334.18	286.23	297.06	11.19	75.73	80.74
M18A1031	431.80	255.51	259.95	10.90	83.35	64.83
M18A0915	282.13	252.64	253.35	12.42	62.27	77.31
M18A1012	260.60	253.519	256.81	10.56	55.42	70.70
M18A1329	276.87	275.685	281.11	11.72	52.27	68.74
M18A1203	233.32	239.643	237.68	13.56	49.82	76.61
M18A1077	300.35	292.034	308.17	13.27	52.69	66.58
M18A1345	264.95	342.628	388.11	14.54	48.22	70.68
M18A1052	551.20	103.352	172.50	12.35	68.23	78.20
M18A1399	241.36	265.602	271.03	16.11	41.57	74.98

Abbreviations: gb, gigabase; kb, kilobase; X, folded of genome coverage.

### 3.2 Comparing optical genome mapping to karyotype

We focus on identifying the structural variations (SVs) detected through OGM, which was reported to be variants with similar karyotype. Compared with the karyotype, eight of 10 samples had variants located on the same pairs of chromosomal arms. In more details, the break area of p and q arms of a sample chromosome detected by OGM was similar to the karyotype, the break area of one arm of three samples (M18A1012, M18A1203, and M18A1345) chromosome was consistent with the karyotype, the break area of two arms of one sample (M18A1077) chromosome was similar to the karyotype, and the break area of one arm of five samples (M18A1031, M18A1329, M18A1203, M18A1052, and M18A1399) chromosome was similar to the karyotype (Table 3). However, the broken area of one arm of six samples (M18A1031, M18A1012, M18A1329, M18A1345, M18A1052, and M18A1399) chromosome was inconsistent with the karyotype (Table 3).

**TABLE 3:** Comparison results of karyotype, FISH, OGM, and CNV

Sample	Karyotype	Bionano	FISH	CNV
M18A0732	*t*(10;11)(p12.2;q12)	*t*(10;11)(q21.1;p12)	*t*(10;11)(p;q)	N
M18A1031	*t*(2;22)(q22;q13)	*t*(2;22)(q14.3;q12.2)	*t*(2;22)(q;q)	N
M18A0915	*t*(Y,19)(q11.21,q12)	(−)	*t*(Y;19)(p;p)	N
M18A1012	*t*(8;15)(p11.2;q26.1)	*t*(8;15)(p21.1;q26.1)	*t*(8;15)(p;q)	N
M18A1329	*t*(2;16)(q13,q12.1)	*t*(2;16)(q14.2;q21)	*t*(2,16)(q;q)	N
M18A1203	*t*(2;9)(p22;p23)	*t*(2;9)(p22.3;p24.1)	*t*(2;9)(p;p)	N
M18A1077	*t*(11;22)(q24;q12)	*t*(11;22)(q23.3;q11.21)	*t*(11;22)(q;q)	N
M18A1345	*t*(9;21)(q21.13;q11.2)	*t*(9;21)(q21.13;q22.11)	*t*(9;21)(q;q)	N
M18A1052	*t*(3;14)(q21;q24)	*t*(3;14)(q22;q31)	*t*(3;14)(q;q)	N
M18A1399	*t*(2;13)(p11.2;q14)	*t*(2;13)(p12;q21.1)	N/A	N

There remaining two samples had various scenarios. There was a translocation in one sample (M18A0732) between the long arm of Chromosome 10 and the short arm of Chromosome 11, which was detected via OGM, but the karyotype analysis showed the chromosomal rearrangement between the short arm of Chromosome 10 and the long arm of Chromosome 11. In the last one (M18A0915), the translocation detected by karyotyping nearby centromeres of Y chromosome failed to be flagged through OGM. Balanced SVs with breakpoints in the region of the repeated around the centromere are expected to escape detection due to missing labels.

In addition, we identified three genes disrupted by OGM rearrangement breakpoints (Table 4), including *WDR33* (WD repeat domain 33) gene (OMIM 618082) in Region 2q of Patient M18A1031, *TMEM37* (betaGal beta‐1, 3‐N‐acetylglucosaminyltransferase 3) gene (OMIM 618831) in Region 2q of Patient M18A1329, and *IL20RB* (interleukin 20 receptor subunit beta) gene (OMIM 605621) in Region 3q of Patient M18A1052. Particularly, *WDR33* had been identified associated with male infertility (Ito et al., 2001).

**TABLE 4:** Gene mapping in OGM SVs breakpoints

Samples	Breakpoint 1	Gene mapping in breakpoints 1	Breakpoint 2	Gene mapping in breakpoints 2
M18A0732	10:55,061,801	–	11:41,869,333	–
M18A1031	2:127,734,067	*WDR33*	22:30,218,554	–
M18A0915	–	–	–	–
M18A1012	8:27,595,163	–	15:91,837,551	–
M18A1329	2:119,435,903	*TMEM37*	16:59,293,070	–
M18A1203	2:35,126,893	–	9:5,237,385	–
M18A1077	11:116809335	–	22:20383088	–
M18A1345	9:71,648,910	–	21:30,439,936	–
M18A1052	3:137,007,599	*IL20RB*	14:84,360,045	–
M18A1399	2:79,080,806	–	13:57,475,856	–

### 3.3 Comparing optical genome mapping to FISH and CNVs

**FIGURE 1:**
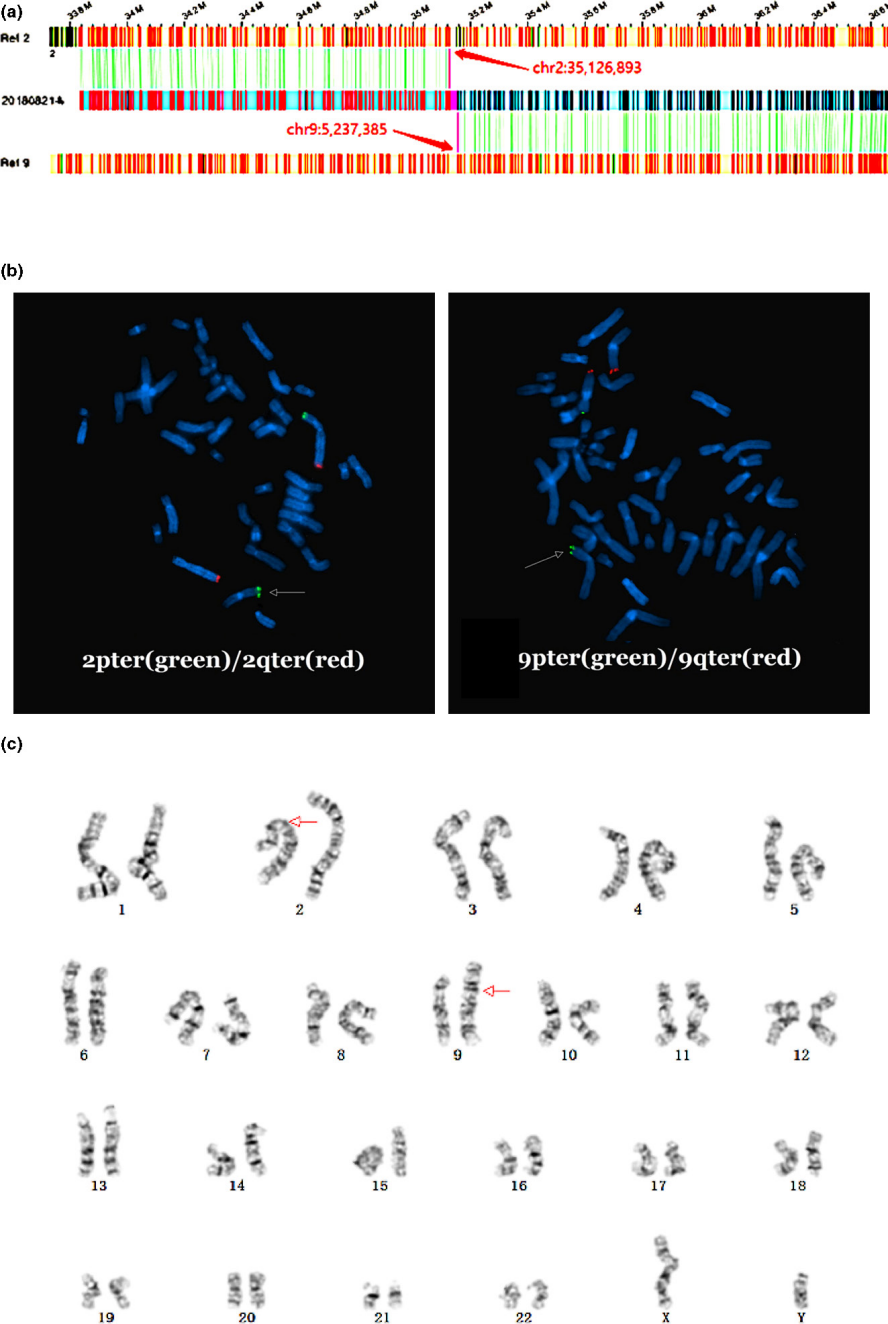
Detection of SVs of M18A1203 via OGM, FISH, and karyotype. (a) OGM indicating the translocation between chromosome 2 and chromosome 9. (b) FISH detection of 2pter (green) in the end of the short arm of chromosome 2, 2qter (red) in the end of the long arm of chromosome 2, 9pter (green) in the end of the short arm of chromosome 9 and 9qter (red) in the end of the long arm of chromosome 9. (c) The translocation between chromosome 2 and chromosome 9 by karyotype analysis

The FISH experiments were designed according to relative events detected through karyotyping. FISH was performed in nine out of 10 samples using available probes related to special chromosomal parts of karyotyping. Seven patients were observed to have SVs detected by OGM that were consistent with those determined through FISH analysis (Table 3, Figure 1). A common reason for the discordance between the two methods in both samples may be the highly repetitive sequences with breakpoints located in the central region of the chromosome. Intriguingly, in M18A0915, both karyotype and FISH identified a translocation between chromosome Y and chromosome 19 (Table 3), which was different in chromosomal arms.

No samples contained CNVs larger than 100 kilobases. We carefully checked the CNVs results around the breakpoint of translocation samples, but did not find any deletions/duplications around the breakpoints, even at 10 kb resolutions.

## 4 DISCUSSION

We have demonstrated that Bionano OGM is an effective approach to detect balanced chromosome translocations compared with traditional methods like karyotyping and FISH. As a new method of nucleic acid sequencing and genome analysis, especially for chromosomal insertions and deletions, OGM is used to analyze multiple DNA fragments simultaneously at a scale of 500 bp as in NGS high‐throughput sequencing, other SVs need to be 30 kb or larger to be detectable (Bionano Genomics, 2018; Dremsek et al., 2021; Jeffet et al., 2021). It makes sure that structural variations can be detected correctly, and more information can be given about the breakpoint position in gene level.

OGM is a new generation of nucleic acid analysis technology developed in recent years that directly labels DNA molecules with fluorescence and allows researchers observe and analyze individual DNA molecules through a fluorescence microscope (Gilboa et al., 2020). In principle, OGM combines some of the features of FISH and NGS techniques. Similar to FISH, OGM uses a huge number of probes evenly distributed on the genome to label chromosomal DNA and observe it under a microscope. While OGM can observe and analyze DNA fragments of only hundreds of kilobases, detection of some repetitive sequence regions such as telomeres and centrioles may still be problematic. Similarly, OGM also simultaneously analyses multiple DNA fragments like NGS, while long DNA fragments in hundreds of kilobases ensure that structural variations can be accurately detected.

Ten cases were enrolled in this study and karyotyping, FISH, and OGM were performed. We found that karyotyping and FISH of nine samples were consistent in chromosome level. The karyotyping results of the remaining case M18A0915 indicated that chromosome Y was fused to chromosome 19 long arm, but FISH suggested that chromosome Y short arm was fused with chromosome 19 short arm. The inconsistency between FISH and karyotype results may be due to poor chromosomal morphology of the cells during the observation period. Also Bionano OGM detected the reciprocal translocation and break regions in nine samples, but the break regions of partial samples were similar or inconsistent with the break regions identified by karyotyping, because karyotyping was rough in identifying break regions. Karyotyping is a morphological analysis technique and has a very low resolution (estimated to be 5–10 Mb on average). There are many factors, such as cell culture time, colchicine concentration and reaction time, temperature and humidity of laboratory environment, and work experience of the technicians, that affect the determination of translocation chromosomes, and may even be missed (Swansbury, 2003), so karyotype results may vary from different laboratory. Therefore, we believe that Bionano OGM has more advantages and high accuracy in resolving chromosomal translocations than karyotyping.

High‐resolution OGM reached 100% concordance compared to karyotyping for all aberrations with non‐centromeric breakpoints (Mantere et al., 2021; Wang et al., 2020). But OGM failed variation detection in one sample in our study, compared to karyotype and FISH. The OGM missed the fusion of chromosome 19 and Y in M18A0915, probably for the following reasons. The recurrent repetitive sequences in chromosome Y, and breakpoint close to centromere, made it difficult to distinguish chromosomes origin of two ends of the breakpoint. Telomere and centromere regions remain poorly comprehended areas of the genome, also data in these regions are scarce and unreliable (Miga et al., 2020). The accuracy and precision of OGM detecting mutations in centromere regions are unknown. Therefore, OGM is not suitable for detecting mutations in the centromere regions, and the abnormality in the centromere region such as Robertsonian translocations cannot be detected by this technique. However, as accurate sequences are obtained for these regions, OGM will gradually improve the accuracy of the detection in these regions. In addition, according to the result of OGM, we rechecked the karyotype and FISH results of the case M18A0732, due to the impact of karyotype quality and staff experience, OGM still showed different result in case M18A0732 with karyotyping of the determination of the long and short arms, which could also be caused by the repetitive region around centromere and the recognition sites for the OGM enzymes to label in those regions.

The CNV test did not provide much useful information and no obvious copy number gains or losses were detected around the translocation breakpoints, so that the patients' clinical presentation was normal. For some patients whose karyotype analysis suggests balanced translocation, CNV detection may not present practical significance. Breakpoints analysis using optical profiles in patients with equilibrium translocations is more meaningful in confirming what is actually happening around the breakpoints.

OGM can provide more detailed information about the location of breakpoints. The DNA was labeled with a fluorophore by the methyltransferase DLE‐1 at the recognition motif CTTAAG, and generated approximately 14–15 labels per 100 kb when labeling human genomic DNA, so OGM has a theoretical resolution of 100 kb (Dremsek et al., 2021). In practical applications, it has a resolution of approximately 100 kb, which is 50 to 100 times higher relative to karyotype analysis. In this study, of approximately 100 kb, which is three genes were found near breakpoints in multiple samples (Table 4), including schizophrenia‐related gene *TMEM37* and atrial septum defect associated gene *WDR33*. Therefore, OGM could provide more genetic evidence in assisting clinical diagnosis, for example preimplantation genetic testing (PGT). If patients select assisted reproductive treatment to overcome fertility, PGT is the best approach for them to pursue select embryos with a normal or balanced chromosome constitution. OGM can provide breakpoint regions and gene disturbances to help them obtain a noncarrier embryo without the balanced translocation. But Bionano OGM is not a convenient way for clinical use nowadays, it needs skilled technician to operate the complex DNA extraction and DNA labeling procedure, it requires high quality sample (2 ml fresh blood or 10^6^ live culture‐cells), especially the puncture samples. Also the cultivation of samples can eliminates the time advantage of OGM over karyotyping. Last, the cost of OGM is much higher than traditional ways and genomic data collection and analysis also may be a limiting factor (Dremsek et al., 2021).

## 5 CONCLUSION

OGM is a new technique that has been developed in recent years. Our research has shown that OGM can be provided more accurate and precise results for the complex region, but the accuracy and precision of OGM detecting mutations need be gradually improved in telomere and centromere regions.

## CONFLICT OF INTEREST

The authors have declared no conflict of interest.

## ETHICAL APPROVAL

This study was approved by the ethics committee of the First Affiliated Hospital of Zhengzhou University (Ethics number: KS‐2018‐KY‐36). All research participants or their legal representatives signed informed consent forms for participation in clinical and genetic research. All methods were performed in accordance with the relevant guidelines and regulations in accordance with the Declaration of Helsinki in this study.

## AUTHOR CONTRIBUTIONS

Peng Dai and Yu Liang designed the study and drafted the manuscript. Jingjing Li and Yanzheng Pei performed Bionano laboratory workflow and analyzed the data. Peng Dai, Xiaofan Zhu, and Zhi Gao collected the sample and participated in all other laboratory workflow. Peng Chen modified the manuscript. Xiangdong Kong oversaw the work and revised the manuscript. All authors have read and approved the final manuscript. If more detailed information is needed, please email Peng Dai and/or Xiangdong Kong.

## Supporting information


Appendix S1

## Data Availability

The reference sequencing data (hg38) in this study were deposited at http://genome.ucsc.edu/index.html. The datasets supporting the conclusions of this article are included within the article and its additional files.
