# Supplementary material for: Evaluation of optical genome mapping for detecting chromosomal translocation in clinical cytogenetics
Source: Mol Genet Genomic Med. 2022 Apr 6;10(6):e1936. doi: 10.1002/mgg3.1936 (PMC9184658; doi:10.1002/mgg3.1936)
Supplement: Supplementary file 1 — Appendix S1 [file MGG3-10-e1936-s001.docx]

**Supplementary Informations**


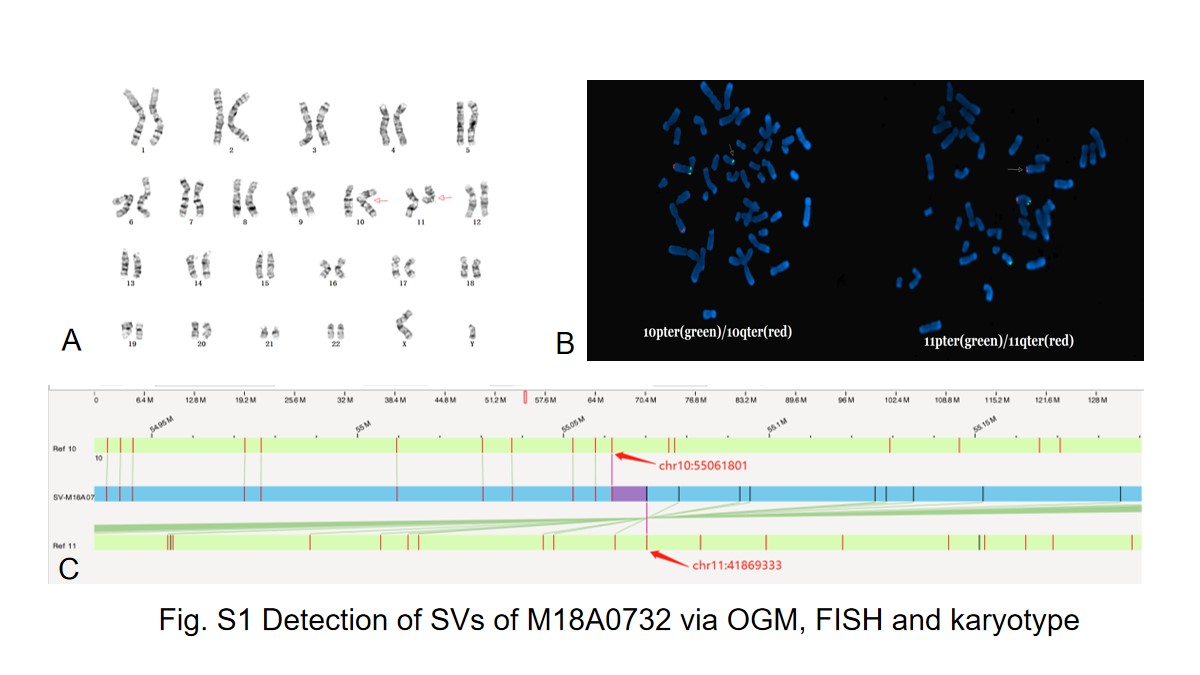


**Fig. S1** Detection of SVs of M18A0732 via OGM, FISH and karyotype. **(A)** The translocation between Chromosome 10 and Chromosome 11 by karyotype analysis. **(B)** FISH detection of 10pter (green) in the end of the short arm of Chromosome 10, 10qter (red) in the end of the long arm of Chromosome 10, 11pter (green) in the end of the short arm of Chromosome 11 and 11qter (red) in the end of the long arm of Chromosome 11. **(C)** OGM indicating the translocation between Chromosome 10 and Chromosome 11.


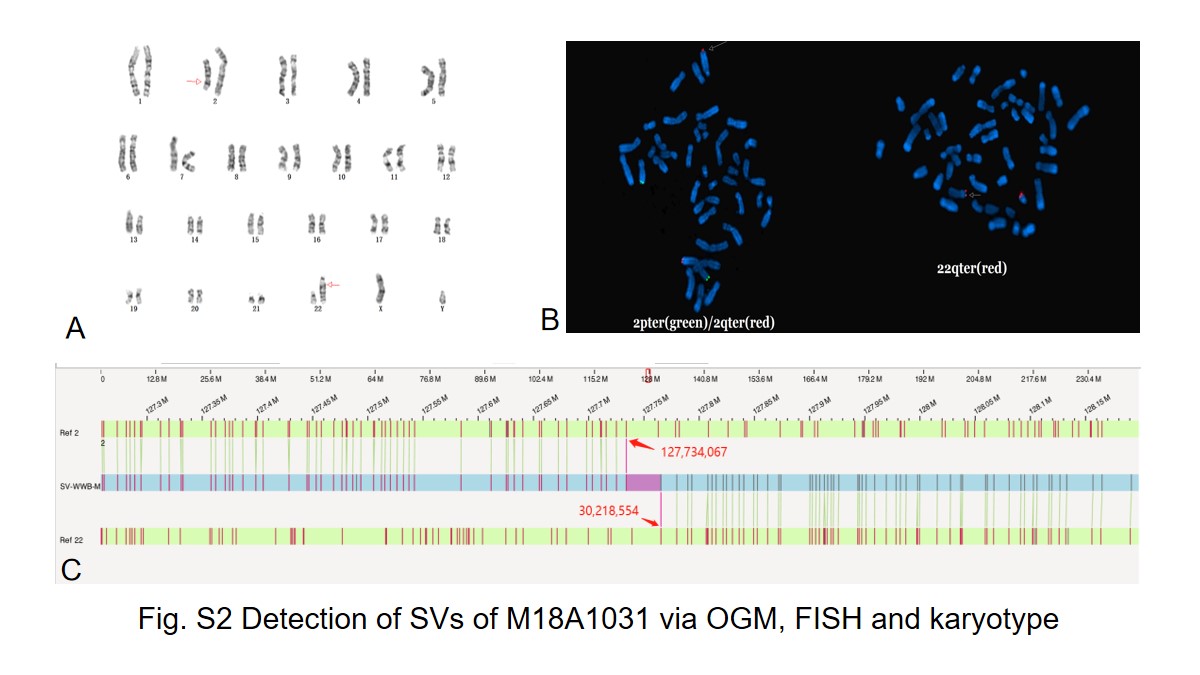


**Fig. S2** Detection of SVs of M18A1031 via OGM, FISH and karyotype. **(A)** The translocation between Chromosome 2 and Chromosome 22 by karyotype analysis. **(B)** FISH detection of 2pter (green) in the end of the short arm of Chromosome 2, 2qter (red) in the end of the long arm of Chromosome 2, and 22qter (red) in the end of the long arm of Chromosome 22. **(C)** OGM indicating the translocation between Chromosome 2 and Chromosome 22.


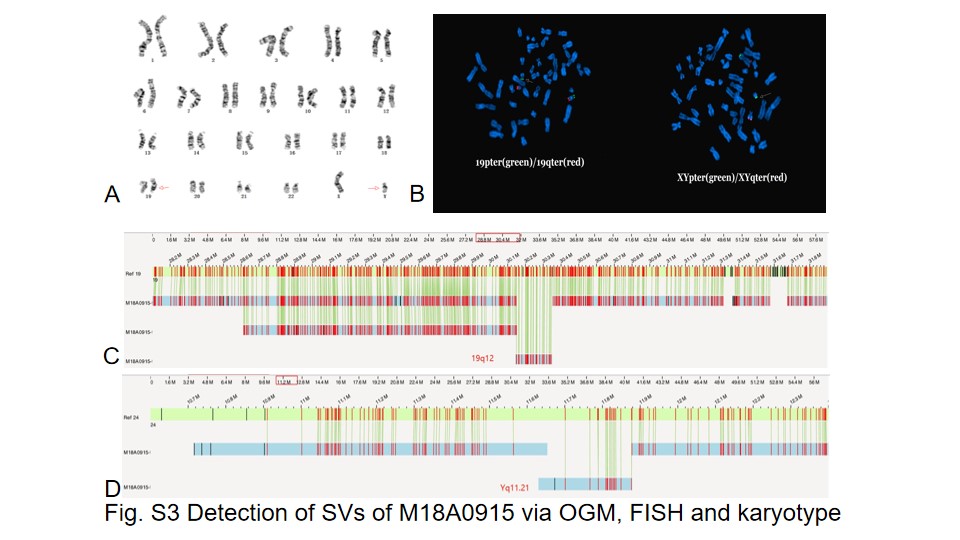


**Fig. S3** Detection of SVs of M18A0915 via OGM, FISH and karyotype. **(A)** The translocation between Chromosome 19 and Chromosome Y by karyotype analysis. **(B)** FISH detection of 19pter (green) in the end of the short arm of Chromosome 19, 19qter (red) in the end of the long arm of Chromosome 19, Ypter (green) in the end of the short arm of Chromosome Y and Yqter (red) in the end of the long arm of Chromosome Y. **(C and D)** OGM failed the translocation detection between Chromosome 19 and Chromosome Y.


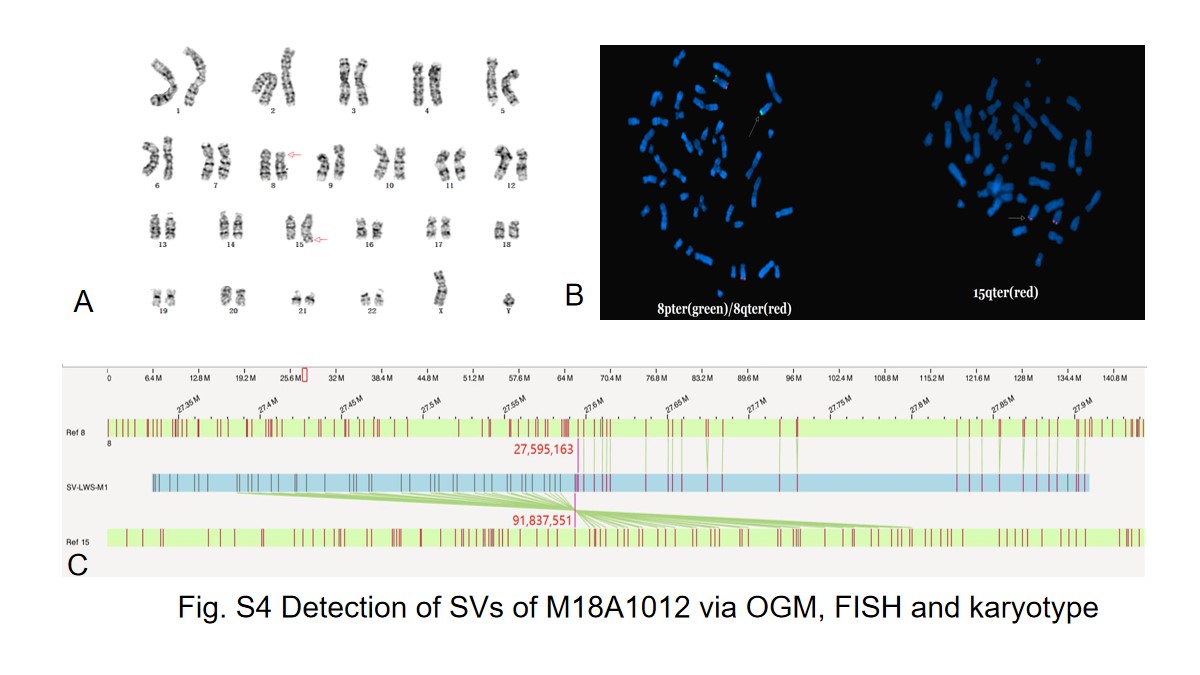


**Fig. S4** Detection of SVs of M18A1012 via OGM, FISH and karyotype. **(A)** The translocation between Chromosome 8 and Chromosome 15 by karyotype analysis. **(B)** FISH detection of 8pter (green) in the end of the short arm of Chromosome 8, 8qter (red) in the end of the long arm of Chromosome 8, and 15qter (red) in the end of the long arm of Chromosome 15. **(C)** OGM indicating the translocation between Chromosome 8 and Chromosome 15.


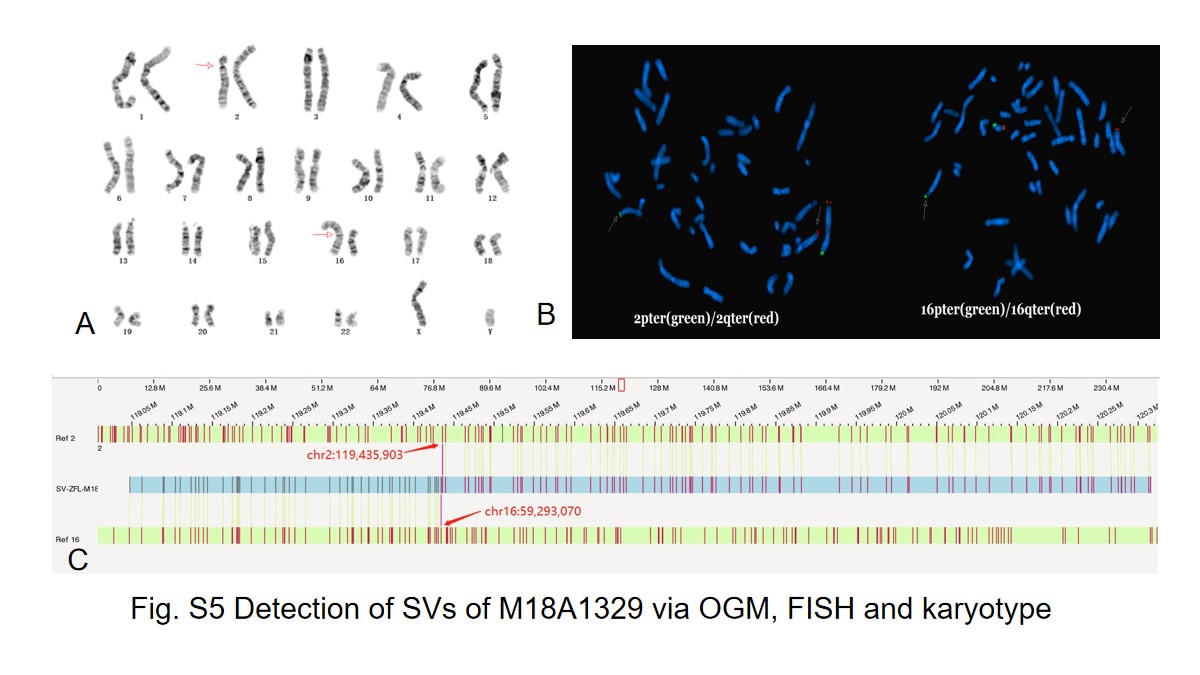


**Fig. S5** Detection of SVs of M18A1329 via OGM, FISH and karyotype. **(A)** The translocation between Chromosome 2 and Chromosome 16 by karyotype analysis. **(B)** FISH detection of 2pter (green) in the end of the short arm of Chromosome 2, 2qter (red) in the end of the long arm of Chromosome 2, 16pter (green) in the end of the short arm of Chromosome 16 and 16qter (red) in the end of the long arm of Chromosome 16. **(C)** OGM indicating the translocation between Chromosome 2 and Chromosome 16.


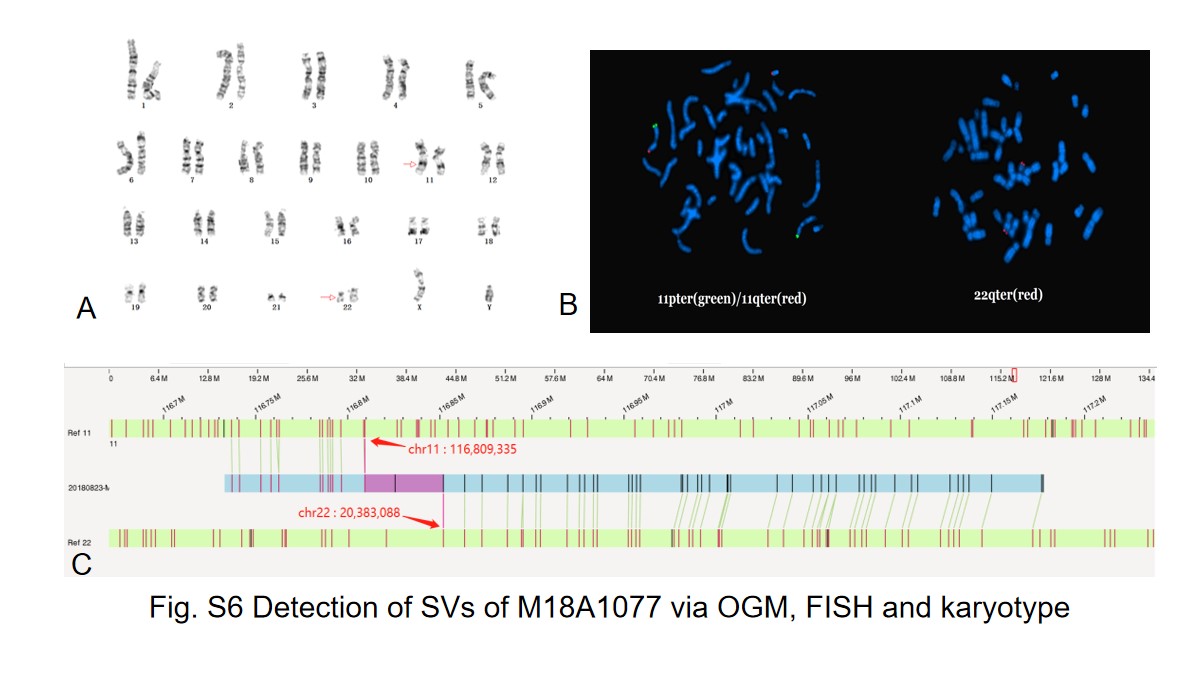


**Fig. S6** Detection of SVs of M18A1077 via OGM, FISH and karyotype. **(A)** The translocation between Chromosome 11 and Chromosome 22 by karyotype analysis. **(B)** FISH detection of 11pter (green) in the end of the short arm of Chromosome 11, 11qter (red) in the end of the long arm of Chromosome 11, and 22qter (red) in the end of the long arm of Chromosome 22. **(C)** OGM indicating the translocation between Chromosome 11 and Chromosome 22.


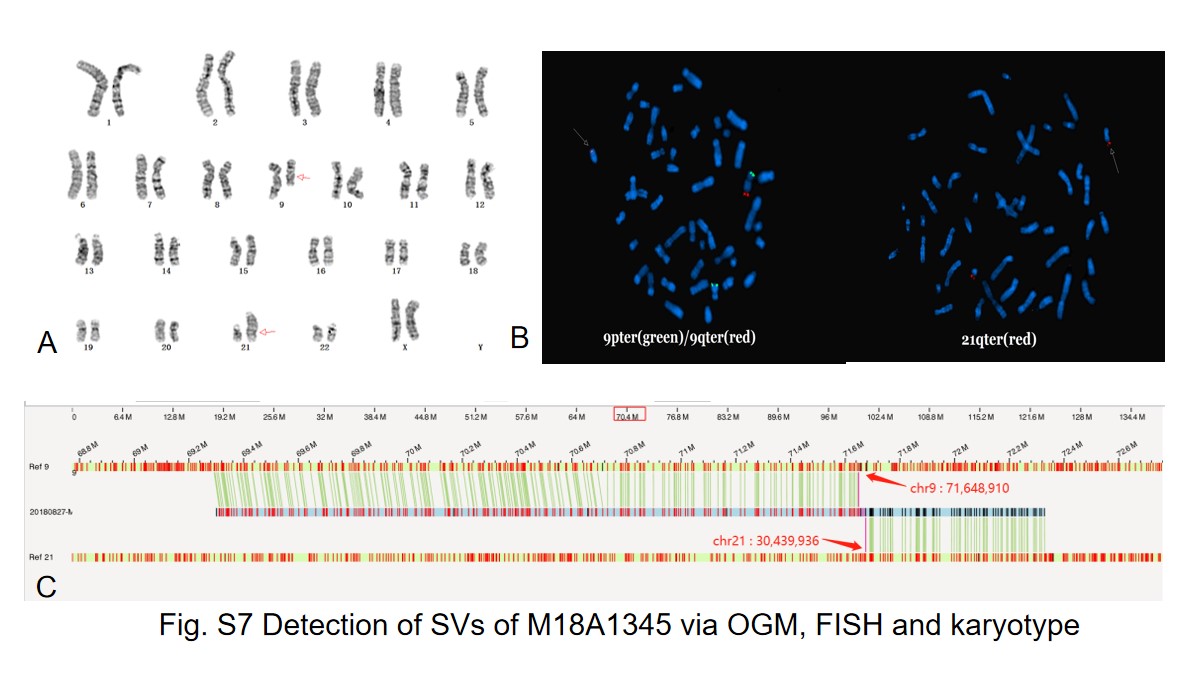


**Fig. S7** Detection of SVs of M18A1345 via OGM, FISH and karyotype. **(A)** The translocation between Chromosome 9 and Chromosome 21 by karyotype analysis. **(B)** FISH detection of 9pter (green) in the end of the short arm of Chromosome 9, 9qter (red) in the end of the long arm of Chromosome 9, and 21qter (red) in the end of the long arm of Chromosome 21. **(C)** OGM indicating the translocation between Chromosome 9 and Chromosome 21.


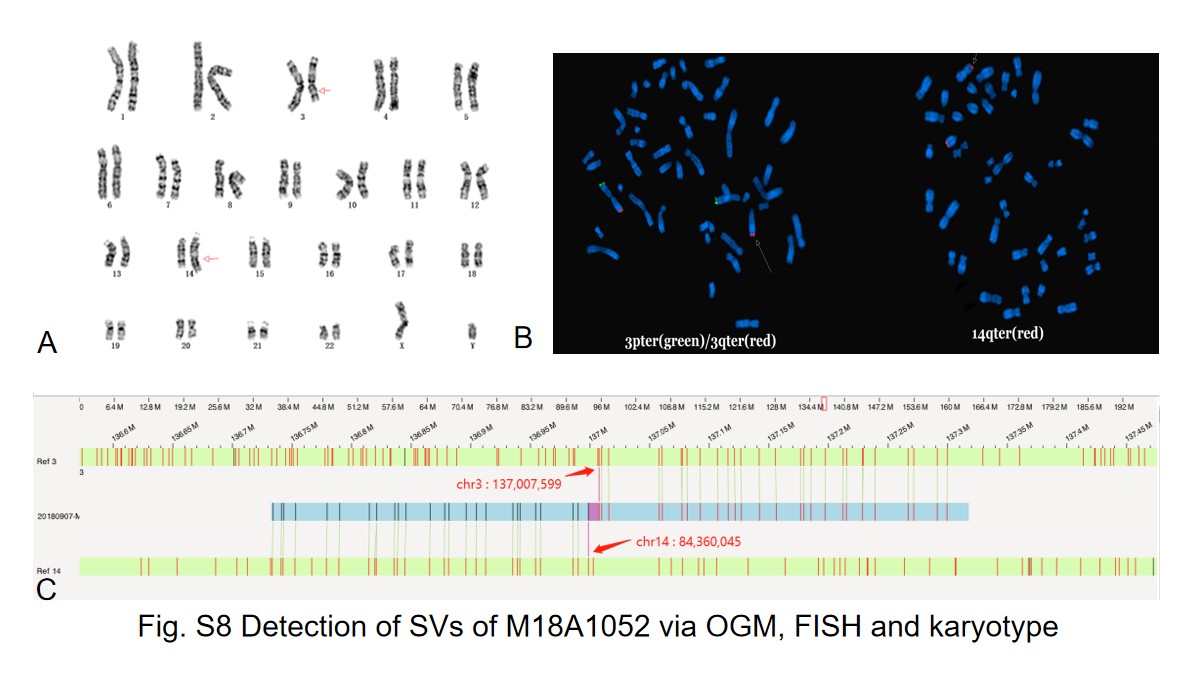


**Fig. S8** Detection of SVs of M18A1052 via OGM, FISH and karyotype. **(A)** The translocation between Chromosome 3 and Chromosome 14 by karyotype analysis. **(B)** FISH detection of 3pter (green) in the end of the short arm of Chromosome 3, 3qter (red) in the end of the long arm of Chromosome 3, and 14qter (red) in the end of the long arm of Chromosome 14. **(C)** OGM indicating the translocation between Chromosome 3 and Chromosome 14.


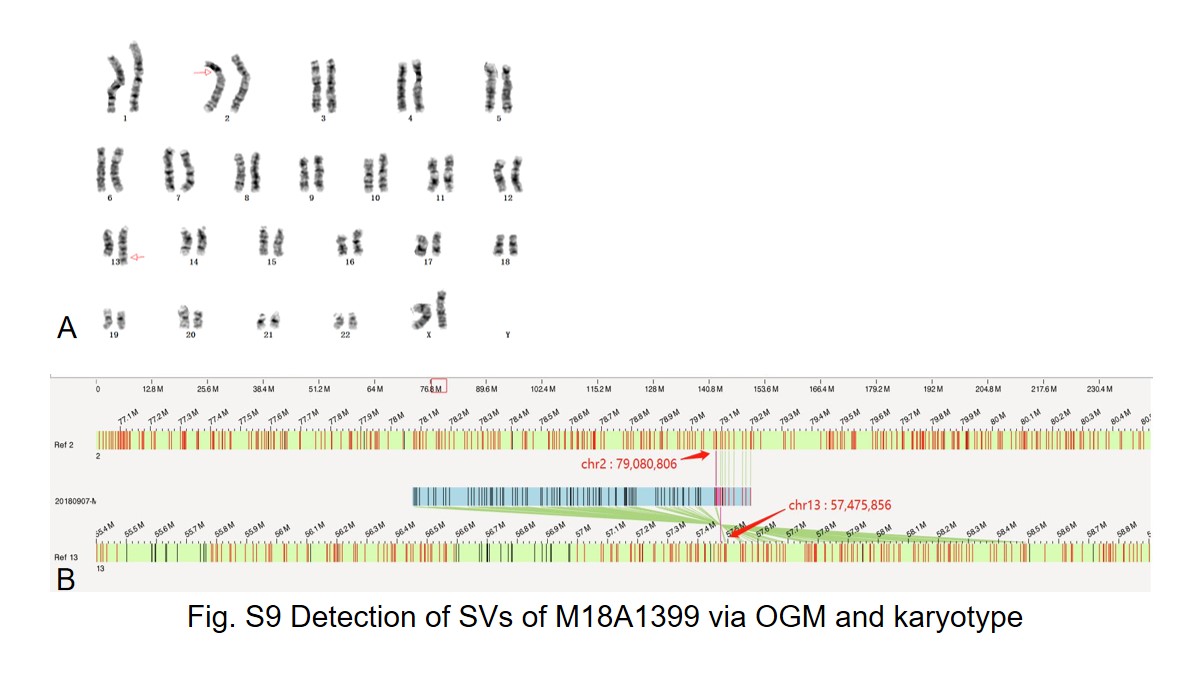


**Fig. S9** Detection of SVs of M18A1399 via OGM and karyotype. **(A)** The translocation between Chromosome 2 and Chromosome 13 by karyotype analysis. **(B)** OGM indicating the translocation between Chromosome 2 and Chromosome 13.
